# Science Policy to Advance a Climate Change and Health Research Agenda in the United States

**DOI:** 10.3390/ijerph18157868

**Published:** 2021-07-25

**Authors:** Jaime Madrigano, Regina A. Shih, Maxwell Izenberg, Jordan R. Fischbach, Benjamin L. Preston

**Affiliations:** 1 RAND Corporation, Arlington, VA 22202, USA; rshih@rand.org; 2 Pardee RAND Graduate School, Santa Monica, CA 90401, USA; izenberg@prgs.edu; 3 The Water Institute of the Gulf, Baton Rouge, LA 70802, USA; jfischbach@thewaterinstitute.org; 4 RAND Corporation, Santa Monica, CA 90401, USA; bpreston@rand.org

**Keywords:** climate change, health, policy

## Abstract

Climate change is thought to be one of the greatest public health threats of the 21st century and there has been a tremendous growth in the published literature describing the health implications of climate change over the last decade. Yet, there remain several critical knowledge gaps in this field. Closing these gaps is crucial to developing effective interventions to minimize the health risks from climate change. In this commentary, we discuss policy trends that have influenced the advancement of climate change and health research in the United States context. We then enumerate specific knowledge gaps that could be addressed by policies to advance scientific research. Finally, we describe tools and methods that have not yet been fully integrated into the field, but hold promise for advancing the science. Prioritizing this advancement offers the potential to improve public health-related policies on climate change.

## 1. Introduction

Climate change is described as the biggest global health threat of the 21st century [1], with some estimates that it could lead to approximately 250,000 additional deaths per year by 2050 [2] and kill more people globally than all infectious diseases [3]. The health risks posed by climate change are wide-ranging, including a wider distribution of infectious diseases, increased water and food insecurity, declining air quality, increased magnitude and frequency of extreme heat and other weather events, and population displacement [1,4,5]. The literature describing these risks has grown immensely in the last decade and has drawn upon historical health effects of weather phenomena associated with climate change. However, several critical research gaps remain, particularly in translating knowledge about the health risks of climate change to effective interventions.

The new Biden Administration in the United States (U.S.) has made tackling the climate crisis a top priority. As such, there have been several notable initiatives enacted, namely through the issuance of executive orders related to climate change, the establishment of new federal offices, and a spending request to Congress to allocate funding towards climate change, with an emphasis on environmental justice. These developments bring significant potential to advance climate change and health policies in the U.S., as well as an opportunity to build momentum and demonstrate the return of these federal-level investments on population health outcomes.

To best harness these investments and achieve the greatest health benefits across a range of health outcomes, it is important to take stock in progress that has been made and explore areas of opportunity. However, because scientific research is dependent upon strategic choices that influence investments, it is important to not only point out research gaps as other syntheses have done, but in addition, to highlight policy choices that could facilitate improved research. In this commentary, we first discuss notable policy trends that have influenced investments in and the advancement of climate change and health research in the U.S., then describe specific remaining research gaps that could be targeted by the renewed enthusiasm for mitigating and adapting to climate change within the current federal policy context, and finally discuss tools and methods that hold promise for closing these gaps in knowledge.

## 2. Policy for Science

Several trends have led to a plateau in climate change and health research within the U.S. The current Administration has an opportunity to change the trajectory of research advancement by addressing these policy issues and ensuring an agenda based on science, not political partisanship. Below, we present trends in three policy areas (funding, coordination, and capacity building) that we believe to be most important in facilitating the advancement of climate change and health research.

### 2.1. Funding for Research on Climate Change and Health

Historically, U.S. federal funding for research on the health effects of climate change has been woefully inadequate. A 2009 review estimated that funding for research on the health risks of climate change—across the U.S. National Institutes of Health (NIH), Environmental Protection Agency (EPA), and Centers for Disease Control and Prevention (CDC)—was less than USD 3 million annually [6]. In 2019, a decade later, annual funding for research on the health impacts of climate change at the NIH had increased to USD 10 million, or USD 235 million when counting the broader category of research on “climate-related exposures and conditions,” much of which is not directly related to climate change [7]. Nonetheless, this only amounts to less than 0.025% of the NIH’s total budget, prompting some to call for the creation of a new National Institute of Climate Change and Health that would be entirely focused on the health impacts of climate change [8].

Ebi and colleagues have pointed out that this lack of funding stems not only from limited resources, but also from the belief that climate change is an “environmental” problem for which primary responsibility for research lies within non-health agencies and institutes, as well as a narrow framing of the health risks of climate change that does not consider systems-based approaches [9]. Further, in the context of climate-related disasters, funding has traditionally focused on emergency preparedness or disaster response and recovery, with health impacts only considered secondarily. However, research tells us that a focus on health is a useful approach to help inform climate change policy and engage the public [10,11,12]. This is because climate change is inherently a health problem—a recent case study of climate-sensitive events in just eleven U.S. states in a single year estimated that the total health-related costs were USD 10 billion [13]. Thus, considering climate change as a public health concern makes a compelling economic case, as well as a humanitarian one. The new Administration’s request for fiscal year 2022 discretionary funding—which includes a USD 100 million increase to NIH’s Climate Change and Human Health program, a USD 100 million increase to CDC’s Climate and Health program, and the establishment of a new Office of Climate Change and Health Equity within the Department of Health and Human Services (HHS)—recognizes the health implications of climate change and is a step in the right direction, although greater efforts are still needed, including in the area of metrics development to measure progress and facilitate accountability.

### 2.2. Coordination across U.S. Agencies and Funding Sources

A long-standing challenge for orienting federal investments in research toward the needs of decision-makers has been coordination among the various research agencies. Since 1990, the U.S. Global Change Research Program (USGCRP) has been the primary vehicle for interagency coordination on climate research. This includes research on the health impacts of climate change, spanning efforts funded by specific agencies as well as more synthetic scientific assessments sponsored by the USGCRP (e.g., [14]). Nevertheless, the U.S. climate change research enterprise has had a historic bias toward the investigation of biophysical processes in the Earth system over understanding the consequences of climate change for human populations [15]. A recent report to the USGCRP therefore recommended agencies boost research efforts that help *“avoid the worst potential consequences of urgent risks to human and natural systems”* as well as manage those risks through adaptation [15]. Population health was specifically identified as one such urgent risk. Moreover, the report recommended organizational changes to USGCRP to (a) enhance agencies’ level of engagement in the program, (b) encourage greater participation of agencies such as the Department of Homeland Security that historically have not participated in USGCRP, and (c) develop public–private partnerships in support of climate change research and science-to-action activities.

Even within the population health and biomedical communities, research efforts are often siloed. Rather than looking at the totality of population health, biomedical research (and funding) is often categorized by the organ system that is affected (e.g., diseases of the respiratory system, cardiovascular system). Recognizing the fault in this framework when dealing with a systemic problem like climate change, with multiple health endpoints, a Trans-NIH Working Group on Climate Change and Health was established over a decade ago. This working group is now co-led by the Fogarty International Center and the National Institute of Environmental Health Sciences, two institutes that do focus on a wide array of health conditions, with broad participation from other NIH institutes [16]. This working group has served to harness expertise from across NIH and provide new perspectives on the climate challenge. Establishing an Office of Climate Change and Health Equity within HHS would do the same across the entire family of health agencies in the U.S. federal government and provide a much-needed coordinating body to bolster this effort of cross-system thinking within health research, practice, and policy.

Of course, responsibilities and resources for research on the health effects of climate change are not limited to HHS; they also take place across multiple other agencies (e.g., the EPA, the National Oceanic and Atmospheric Agency). To reduce the risk of duplicative efforts, ensure a harmonized climate change and health research agenda, and broker partnerships across various organizations that contribute to climate change and health research, a strong coordinating body is needed. The newly established White House Office of Domestic Climate Policy and National Climate Task Force reflect the Administration’s intent to take a “whole-of-government” approach to the climate crisis. With representatives from 21 federal agencies and departments, the task force represents a prime opportunity to elevate and direct resources to addressing the health impacts of climate change. Decades of research on the social determinants of health have shown that population health is just as much, if not more, a product of housing, food, transportation, education, emergency management, and energy systems as it is a product of the healthcare system [17].

### 2.3. Capacity Building and Data Integration

Research to characterize the full range of climate change-related health impacts has been limited by fragmented and inconsistent data and health surveillance systems across the country. While multiple efforts have been made to strengthen the capacity of public health surveillance systems to support health-related adaptation to climate change at the federal, state, and local levels, these efforts have largely operated independent from one another, without the benefit of a shared strategy [18]. Consequently, research is limited in its ability to comprehensively assess health risks, systematically assign economic value to that risk, and ultimately, inform policy [19]. Although there have been recent advancements in climate-health valuation case studies [13,20], data gaps and limitations preclude systematic assessment. Further, data on exposure to climate hazards vary in their precision, accuracy, and availability to health professionals, necessitating cross-sector partnerships with other scientists, practitioners, and members of the public [21]. Data coordination and the promotion of open data and science could engender trust and expand opportunities for more researchers to engage with the creation, utilization, and exchange of datasets used to support a climate change and health research agenda.

To properly conduct surveillance and fill in key data gaps on climate change and health, the U.S. needs a well-resourced workforce not just at the federal level, but also at state and local levels. The COVID-19 crisis has made it abundantly clear to all what many in public health have known for a long time—years of budget cuts and chronic underfunding have left our public health infrastructure ill-equipped to maintain fundamental responsibilities, let alone respond to the intense needs of a crisis situation [22]. The U.S. CDC has operated the Climate-Ready States and Cities Initiative (CRSCI) to build local capacity to assess and respond to the health impacts of climate change since 2010. With minimal funding, this initiative has enabled public health departments in 16 states and two cities to more fully integrate health into broader climate planning [23]. However, to ensure a consistent national strategy, funding for this program needs to be greatly increased to allow broader participation by state and local entities.

In addition to building capacity within the U.S., public health capacity-building for climate change outside of U.S. borders will be of primary importance in the future. Health assessment research does not regularly consider negative externalities of non-U.S. disease burden beyond countries that share physical borders, economic interdependencies, national policies, and programs to mitigate or adapt to climate change effects that are not funded by U.S. development agencies, or the robust research on climate change and health conducted outside of the U.S. In order to effectively combat health inequity and transform resilience both within the U.S. and globally, international coordination is critical for identifying the distribution of risks and disease-burden, as well as sharing experience of effective adaptation and mitigation [24].

## 3. Science for Policy

### 3.1. Research Gaps

As we look to the next decade and beyond, health researchers have a crucial role to play in informing choices and understanding trade-offs of different climate risk management approaches. While there has been important knowledge generation on the risks of some of the most common climate-sensitive health outcomes, crucial gaps remain in our understanding of the full range and magnitude of adverse health impacts associated with climate change and the effectiveness of different strategies to protect health. These research gaps are described more fully below.

#### 3.1.1. Climate-Sensitive Health Outcomes and Economic Burden

Projecting how climate change will either exacerbate existing health threats or give rise to new ones can inform decision-making for public health policy priorities [25]. The Fourth National Climate Assessment, for example, summarizes climate-sensitive health outcomes including increased mortality and injury from more frequent and severe weather-related events and temperature extremes; more respiratory, cardiovascular, and allergic reactions arising from worsening indoor and outdoor air quality; more vector-borne diseases due to changing vector ecology (and footprint) brought about by rising temperatures and changing precipitation patterns; mental health consequences resulting from severe weather-related events (e.g., hurricanes) or prolonged climate patterns (e.g., drought); and compromised nutritional status due to greater food insecurity arising from the effects of rising global temperatures on crop productivity and food distribution systems [26]. Evidence for these effects is based on research using data on the “baseline” incidence or prevalence of relevant health conditions, the expected change in weather exposure due to climate change, and the exposure–response function based on historical effects of weather [27]. The empirical evidence to support these exposure–response relationships is greater for some health outcomes than for others, constraining our ability to project future impacts across the entire spectrum of health outcomes. Therefore, the first data gap we note relates to the breadth of climate-sensitive health outcomes that have been the focus of research.

Although a large body of evidence has accumulated estimating the exposure–response relationship between weather events and some health outcomes, others have been less studied. For example, mental health effects (e.g., suicidality, anxiety, or major depressive episodes) that can occur after population displacement from extreme weather events, as well as neurotoxic effects of air pollution and, more broadly, health effects from air pollution specific to wildfires, which may be different than the impacts that result from air pollution from industrial or mobile sources, are areas where further research is still needed. Further, much of the research to date on climate-sensitive health outcomes has been conducted in adult populations, but there is growing evidence of a wide range of impacts on children, which deserves further attention [28].

Along with characterizing the full range of climate-sensitive health outcomes, it will be important to simultaneously estimate the economic costs associated with these health risks. Although this is a critical step to inform policy, few studies have provided information on the health-related costs of failing to respond to climate change, and in fact, most estimates of economic costs related to climate change omit health-related costs [19,29]. Recent case studies have demonstrated that health-related costs of climate-sensitive events are substantial [13,20] and, thus, are an important factor that is absent from the current policy debate.

#### 3.1.2. Attributing Health Effects to Long-Term Climate Change

Enhancing understanding of the health impacts of climate change is also dependent upon overcoming challenges regarding the detection and attribution of a climate change signal in health outcome data. Although methods for detection and attribution have been applied in multiple contexts [30], few studies have applied them in the health context [31,32]. Though, one recent study provided evidence for the role of climate change in heat-related mortality over the last 30 years in the context of 732 locations across 43 countries, including the U.S. [33]. Whether a clear signal emerges for other health outcomes and when will be contingent on the rate and magnitude of future climate change, demographic changes, as well as investments in adaptation and resilience [34]. Improving the assimilation of health outcome data into model-based estimates of future climate change consequences can help constrain future projections.

#### 3.1.3. Effectiveness of Interventions to Blunt the Health Effects of Weather

Accurately projecting the effects of climate change on human health not only requires an assessment of disease burden across diverse populations, but also an understanding of the types of interventions that can be employed to lessen the acute health effects of weather, a quantification of how effective these interventions are, and an assessment of how acute weather effects and adaptive capacity will jointly translate to health burden from and adaptive capacity to long-term changes in climate.

Empirical evidence on the effectiveness of specific intervention strategies for adaptation is needed to project future health impacts of weather events and help decision-makers assess alternative policy options [35,36]. Such evidence, to date, has been limited by the narrow scope of strategies assessed (e.g., heat adaptation plans and alerts for extreme heat events) [37,38,39], methodologies not adequate to infer causal effects [40], and an incomplete accounting for the full range of health co-benefits that could result [41]. A systematic review of the effectiveness of heat adaptation strategies, for example, noted that most studies focused on community heat warning systems, with limited or no attention to other measures such as air conditioning or infrastructure enhancements such as green infrastructure or white roofs [42]. Relatively few studies have evaluated and compared the effectiveness of the wide range of interventions that have the potential to blunt the adverse health impacts of extreme weather and climate variability, even though this is precisely the type of information that decision-makers need to make investment decisions [43]. The lack of studies on effectiveness can partially be explained by limitations in the availability of spatially and temporally resolved health outcome data that would be necessary to evaluate adaptation interventions for their health effects. However, with more and more locales enacting policies and interventions to address climate change and with data to estimate spatially resolved weather patterns becoming increasingly available, evaluations (which include an assessment of health impacts and co-benefits) should be prioritized as part of their implementation.

Even when empirical evidence on interventions is available, more rigorous evaluation is needed. Many prior studies have relied upon descriptive pre–post approaches (e.g., comparing deaths on extreme weather days before and after the implementation of a specific intervention), which cannot adequately account for confounding due to socioeconomic factors or secular healthcare, demographics, and mobility trends [42]. Furthermore, while it has long been recognized that well-designed adaptation strategies can both directly reduce health risks from climate fluctuations and optimize indirect health co-benefits [44], many studies have focused on the health co-benefits of mitigation [45,46]; however, few studies have examined the health co-benefits of adaptive intervention strategies [41]. For example, when urban green spaces have been assessed for their ability to combat the heat island effect [47], their total health benefits and co-benefits (e.g., an increase in physical activity of nearby residents) have not been captured systematically. Pursuing rigorous assessment of co-benefits could help lead to improved, evidence-based adaptation strategies.

#### 3.1.4. Short-Term Weather-Related Health Effects versus Long-Term Climate Change Impacts

Severe weather events (e.g., heat waves, hurricanes) often occur as initial shocks, which subsequently affect morbidity and mortality. The current literature, which has relied on exposure–response relationships from historical data, has made an important contribution by enumerating and quantifying a range of health effects from these types of acute events. However, it is important to note that most studies to date have not fully addressed the health impacts of long-term climate stressors—for example, the effects of gradually rising temperatures associated with climate change. The distinction between acute weather events and gradual climate change is an important one. On one hand, the frequency of short-term events may make them relevant to long-term projections that predict increased frequency. On the other hand, short averaging periods that describe only acute incidents may not reflect systemic responses to long-term change, nor can they account for either autonomous (e.g., physiologic acclimatization or behavioral changes) or planned (e.g., heat health warning systems) population adaptation [30,48]. Assessing the effects of long-term change requires complete, longitudinal datasets that permit observations of changes in both risk of exposure and health outcomes, as well as models that can project plausible future climate changes and associated impacts across appropriate time horizons and geographic scales. Though often not available, such data could also facilitate the understanding of risk from compound climate events, which would help to better characterize the potential effects of such high-impact events [49].

#### 3.1.5. Accounting for Adaptation in Health Studies

Even in studies that have examined long-term data series, critics have noted a failure to adequately account for long-term population-level adaptation [50]. Astrom and colleagues investigated heat-related mortality in Stockholm during two periods, 1900–1929 and 1980–2009, and concluded that mortality rates in the latter period were double what would have been observed in the absence of climate change [32]. However, others have argued that had population heat sensitivity remained constant (i.e., absent an adaptation response or increase in population resilience due to favorable health and socioeconomic development), the effect would have been much larger [50]. A limited number of studies that have been able to examine mortality data over decades have reported a decline in heat sensitivity [51,52]. However, as Hondula and colleagues conclude in their review of the literature, this stands in contrast to the projection-based body of literature, which predicts a substantial increase in heat-related mortality in the future [53]. Further, many projections that leverage long-term data series do not acknowledge the potential for threshold effects, whereby sensitivity can be latent or even be exacerbated after a given threshold (e.g., heat-related morbidity would be sensitive to a wet-bulb temperature of 35 °C) [54].

Increasing the accuracy of long-term projections of climate-sensitive health outcomes requires proper attribution of the health effects that are due to long-term climate change and the incorporation of autonomous and planned adaptation (and maladaptation) into the analyses to the extent possible [55]. To date, there have been limited attempts to do so through statistical approximations, with little empirical evidence to confirm the magnitude of adaptive response in these assumptions [56]. A partial solution would entail evaluating autonomous adaptation and relying on assumptions for acclimatization or behavior changes. A better solution would be to use empirical evidence (obtained from intervention effectiveness studies described in the previous section) to improve assessments of planned adaptation; however, to date, such data have been limited or non-existent [41]. For example, declines in heat sensitivity have been attributed to the increased prevalence of air conditioning, the introduction of heat warning systems, and longer life expectancies due to socio-economic development and epidemiologic transitions, among others. However, with few exceptions [57], there has been almost no direct analysis to quantify the intervention effectiveness of these specific measures. Beyond effectiveness, to date there have been no systemic empirical analyses that we know of which look at the affordability and availability of adaptation interventions, and consequently their implications for inequities in climate-sensitive disease burden over the long term.

Due to the diffuse and context-specific nature of mitigation and adaptation, there will also continue to be a large degree of uncertainty associated with any given strategy to project health impacts, even with an accumulation of empirical data. Remais and colleagues, for example, have discussed the following uncertainties when modeling health co-benefits of emissions-related mitigation activities that are also relevant when evaluating adaptation strategies: (1) spatial and temporal changes in health-relevant exposures; (2) the time response of the health effects due to exposure changes; (3) alternative interventions in terms of their health effects across populations and time scales; and (4) the assumed time course of future disease-specific burdens in the absence of intervention [58]. Therefore, it is essential that any evaluation of the health effects of long-term climate change include an explicit characterization of uncertainty, particularly as many long-term trends—frequency and intensity of extreme weather events, frequency of compounding events, and susceptibility and resilience of populations—continue to evolve [59].

#### 3.1.6. Centering Equity in Climate Change and Health Research 

It is well known that the health impacts of climate change affect different populations differently and the largest burden has fallen on marginalized communities, both within the U.S. and around the world. In the U.S., evidence has shown a higher burden of climate-sensitive outcomes in communities of color and low-income populations. Like most of the public health literature, studies of climate change and health have relied on simple demographic descriptors, including race, to assess which populations are most at risk. However, there is a growing call within research and practice to recognize that race is a social construct and that the continued use of this construct obfuscates the role of racism in determining health [60]. A serious examination of racism as an explanation for racial disparities is particularly relevant for climate-sensitive health outcomes because of the interactions between policies and social, natural, and built environmental systems that result in unequal environmental burdens for diverse populations [61].

It is imperative that future research on climate change and health be conducted with equity as a central tenet. Approaching climate and health research from this perspective means a deep consideration of upstream drivers of health outcomes, including systemic and structural factors, with a vision toward justice. In this way, researchers can look not just at race or socioeconomic status as the distinguishing factors that determine disparate health outcomes, but rather, the upstream policies and practices that have led to unequal burdens in particular groups. As there is a growing recognition that measures to adapt to climate change may sometimes be maladaptive and worsen inequity, it is all the more important that equity is centered in research and policy approaches to mitigate the health impacts of climate change [62].

#### 3.1.7. Integration across Time Horizons and Spatial Scales

Despite a substantial body of literature on the health effects of mitigation strategies [63,64,65,66,67,68], there are few examples of integrated analyses of mitigation and adaptation strategies. In some cases, these can lead to explicit tradeoffs [69]. For example, the most effective adaptation strategy for preventing heat-related morbidity and mortality is to increase access to air conditioning [57]. However, without sufficient renewable electricity generation, such a strategy will result in an increase in greenhouse gas emissions, directly conflicting with mitigation goals.

The complexity in addressing mitigation and adaptation in an integrated way is driven, in part, by different temporal and spatial scales. While adaptation strategies are typically more focused on the near-term (years to decades), mitigation actions focus primarily on longer-term solutions (decades to centuries). In addition, adaptation is implemented on a local scale with a local impact, while mitigation strategies are intended to have a global impact and are more likely to be implemented at national and international scales. Nonetheless, the downstream effects of mitigation and adaptation can interact with each other regardless of the level at which they are implemented [69]. Identifying the right balance of mitigation and adaptation strategies will depend on methodological tools that can incorporate long and short time-horizons, as well as systems frameworks, into the evaluation of alternative policies. A recently developed conceptual framework [70] presents how a variety of interventions (mitigation and adaptation) affect health outcomes through different pathways and may provide a starting point to addressing this complexity.

### 3.2. Promising Tools and Methods

Although we have outlined several prominent research gaps in the climate change and health literature, there are also several methodological tools that have not yet been fully integrated into the discipline. The incorporation of these tools into the next stage of preparing for health risks from climate change could serve to advance science, practice, and policy.

#### 3.2.1. Quasi-Experimental Designs

Quasi-experimental study designs are used to estimate the magnitude of causal effects on health that are attributable to exogenous variation in the exposure of interest; however, the assignment of exposure groups is not under the control of researchers [71]. While there is no definitive list of quasi-experimental study designs, frequently captured within this group are natural experiments, instrumental variable analyses, regression discontinuity analyses, interrupted time-series, and difference studies (controlled before-and-after studies, difference-in-difference studies, and fixed effects analyses of panel or longitudinal data) [72].

Although quasi-experimental studies are not necessarily of higher quality than non-experimental studies, in some cases, it may be easier to verify their identifying assumptions for causal inference. For example, in non-experimental studies, all potential confounders (time-varying and time-constant) must be measured and controlled for valid causal inference. Verifying that this assumption is met is extremely difficult and often impossible. For quasi-experiments, the set of identifying assumptions may also be very difficult to verify, but in some cases, can be less onerous than non-experimental studies. For instance, in the set of difference studies, one must only assume the absence of bias from time-varying confounders. In the case of assessing the effect of a heat adaptation plan, for example, one would only need to control for other time varying policies, programs or factors that might affect the association between heat and mortality in the exposed and comparison locations (e.g., cities or neighborhoods). By contrast, in a non-experimental study comparing the health impacts of various heat policies across locations, one would need to control for all factors that varied between the exposed and comparison locations, including differences in demographics and other time-constant characteristics. Other types of quasi-experiments have design-specific identifying assumptions that must be verified. We are encouraged that there have been recent additions to the climate and health literature using a quasi-experimental approach [40]. As more and more locales adopt adaptation strategies, there will be increasing opportunities to apply quasi-experimental designs in their evaluation.

#### 3.2.2. Detection and Attribution Analysis

Detection and attribution analyses permit an understanding of the role of climate change in current extreme weather events. The research community recognizes the importance of this type of work in building the evidence base for climate risk management and to inform accurate projections of how the health effects of climate change can be minimized through climate mitigation. To date, however, there have been very few examples of detection and attribution methods applied to health research [30,31,32,33]. Ebi and colleagues have developed causal chains for case studies for several extreme heat events to demonstrate how impact attribution can be used to inform health system preparedness and response interventions [73]. The ability to conduct detection and attribution analysis relies on knowledge of appropriate exposure–response functions and, thus, again points to the need for further research across a wide range of climate-sensitive health impacts [55]. Although detection and attribution methods have been rarely applied to health research, and approaches vary widely, early case studies demonstrate their suitability and indicate that they will likely play an important role in the next phase of climate change and health research.

#### 3.2.3. Decision Analysis

Decision-making for climate change must account for uncertainties associated with different model predictions and projections [74]. Due to these uncertainties, the varied temporal and spatial scales that climate decisions are made on, as well as the competing resource demands that decision-makers face, tools and frameworks are needed to support complex, multidimensional decisions to be made with respect to climate change policy. We highlight two here.

Multi criteria decision analysis (MCDA) is a quantitative method that can be used to rate and compare alternative options against a range of criteria. This can take the form of subject matter experts and decision-makers who subjectively rate all options as well as a median, lower, and upper quartile for each rating in order to inform distribution functions for each of the ratings. MCDA involves the formulation of the problem statement (including the definition of decision options and criteria), building an evidence matrix per criterion, determining the relative importance (weighting) of each criterion and the integration of the evidence matrix and weighting to determine an overall score. The benefit of using MCDA to support public environmental health decision-making has been demonstrated in the context of MCDA to evaluate the impact of different types of interventions to reduce the health burden of four different environmental health hazards, ranging from vehicle emission controls to reduce ambient air pollutants to encouraging active transport and increasing access to green spaces [75].

Robust decision-making (RDM) is a methodology for decision-making under deep uncertainty (DMDU) that allows stakeholders to consider necessary trade-offs in allocating resources across a broad portfolio of potential interventions. Although quantitative analysis is needed for sound policy decisions, traditional quantitative analysis provides information about the future by making predictions. Since predictions can be wrong, and decision-makers are aware of that reality, quantitative analysis can be discounted. Instead of using models and data to predict the future, the RDM methodology runs models on hundreds to thousands of different sets of assumptions to describe how plans perform in a range of possible futures. Visualization and statistical analysis of the resulting model outputs help decision-makers understand future conditions under which their plans perform well and those under which their plans perform poorly, guiding them to make their plans more robust [76]. RDM has been applied to a wide variety of policy areas, including resource planning for climate adaptation, coastal resilience planning, and national security. Common uses of RDM for these topical areas include prioritizing policies across time, ranking policies by cost effectiveness, ranking policies by alternative criteria, and understanding complementarities and substitutability (i.e., tradeoffs) across policies. Due to the deep uncertainty associated with the public health impacts and appropriate responses to climate change, RDM is uniquely suited to tackle this challenge.

#### 3.2.4. Systems Approaches

Climate change, like other complex public health problems, is socially complex and difficult to define, has local and global impacts, and requires interdisciplinary solutions [77]. Burke and colleagues called for a systems approach to look at such problems holistically [78]. Similarly, a recent report to the USGCRP on research priorities for the next decade recommended a systems-based research agenda that integrates the natural and social sciences [15]. The basic tenets of complex systems theory, such as non-linearity, adaptiveness, and connectivity, make it well-suited to apply to climate change and health, which must account for dynamic context influencing operations, as well as interactions among system components including the natural environment, infrastructure, economic and social behavior patterns, human subpopulation effects [79], and between a given system and the larger environment [80].

Moreover, a systems approach can aid our understanding of system implications and the risk of unintended consequences that may occur when integrating mitigation and adaptation strategies [81]. Although cities do not exist or act in isolation of each other, with the rapid growth of global urbanization, one approach that Da Silva and colleagues [82] argue for is an urban systems approach that improves upon traditional risk assessment based on spatial analysis and climate projections. Urban systems approaches can be applied to health risks to estimate how a city’s public health, emergency management, infrastructure, and vulnerable socio-demographic groups behave and fluctuate as a complex system across multiple temporal and spatial scales in the face of short-term and long-term climate change effects and therefore how to best prepare for them. Systems analysis and complexity studies can be leveraged to demonstrate how structural factors such as technology and equity of information contribute to differential levels of environmental exposure, and how these dimensions may impede or advance climate change and health policy goals [83].

#### 3.2.5. Community-Partnered Research and Citizen Science

Engagement with stakeholders, including policymakers, health professionals, and the public, is a critical part of preparing for and responding to the health risks generated by climate change. Most crucially, place-based and community-partnered research works to ensure the co-development of climate change policy goals for (often disproportionately) affected populations [84]. This is particularly important given the social, economic, and psychological values of each stakeholder, notwithstanding their understanding or perceptions of climate change [79]. Community-partnered research, when applied correctly, often requires that researchers and community members design and validate research and programming to ensure local relevance, feasibility, and that the proposed approaches are desired by and in line with local communities. Consequently, when research is not community-partnered, the unintended consequences could include low levels of buy-in, misappropriation of resources to not address local issues of priority, disillusionment or skepticism in research, or non-compliance where benefits to communities are not immediately evident. When communities drive their own research agenda and stakeholders facilitate and elevate research, community-partnered research has been demonstrated to effectively promote long-term adaptive capacity [85].

Citizen science, or scientific research that is conducted in whole or in part by nonprofessional scientists, has become increasingly popular in the last few years. Similar to community-partnered research, citizen science supports local residents to engage in scientific research activities, with levels of engagement ranging from leading the initiative and asking the research questions, to supporting more traditional academic research initiatives by supporting and contributing to data collection [86]. Citizen science is well suited for public policy research related to climate change and health risks because it engages affected populations from the start [87]. The internet, mobile technology, and geographic information systems have enabled citizen scientists to collect large volumes of useful data and have contributed to the increased use of citizen science in a wide variety of scientific endeavors. For example, citizen science has commonly been used to enhance public health emergency preparedness during and following extreme weather events [88]. Despite the rise in citizen participation in policy research, the full potential of citizen science has not been fully realized because of challenges related to the translation and use of the data, the sustainability of citizen science efforts, as well as data quality concerns [86]. Critical to improving equity in climate and health plans, an additional concern is that citizen science participants may not necessarily reflect the broader population, therefore inadvertently exacerbating inequities in climate change agenda-setting [89]. Further research is needed to determine how citizen science can optimally be used to help populations mitigate and adapt to the health risks of climate change, as well as ways to include disproportionately affected communities in constructing and executing their own locally relevant research agenda.

## 4. Conclusions

In summary, there is a policy window in the U.S. to advance the science on climate change and health, while centering equity in this work. Several critical questions and uncertainties remain to better understand and help prevent or reduce the health risks associated with climate change, and to better understand the effectiveness of different intervention strategies. The current focus on acute health effects from weather events limits our ability to understand longer-term, population-level health impacts and adaptive change. Large uncertainties in the estimates of the health impacts of climate change exist because of the dearth of research evaluations on a range of adaptation strategies, perhaps somewhat attributable to the diffuse nature of adaptation. Mitigation and adaptation strategies interact to reduce the health impacts of climate change, yet few studies have integrated the analysis of combined strategies. Such studies are critical given the sometimes-conflicting goals and spatial and temporal differences between these two approaches. These gaps in knowledge make it even more difficult for decision-makers to act on climate change despite the large toll that climate-sensitive health risks have taken to date and will continue to take in the future. Research that provides evidence to close such gaps can help policymakers weigh the costs and benefits of interventions and develop clear targets and measures of accountability for climate mitigation and adaptation. 

Despite these gaps, there are tools and methodologies that climate and health researchers have yet to fully harness. Future epidemiologic studies should focus on climate-sensitive outcomes that have been less studied and incorporate economic valuation. Researchers should collect longitudinal data related to climate variability and health outcomes in specific locations and use quasi-experimental and other rigorous study designs to evaluate the effectiveness of intervention strategies and characterize the uncertainty in those estimates. Climate change and health researchers may benefit from applying detection and attribution analysis to better quantify the benefits of mitigation actions. Decision analysis frameworks have been applied to the evaluation of climate change mitigation and adaptation but not specifically to their health impacts. Decision analysis methods such as MCDA and RDM, combined with systems approaches, can provide more robust information for decision-makers who must weigh the uncertainties in climate-sensitive health risk projection and its multi-sector interdependence when considering policy options. Lastly, public engagement methods such as community-partnered research and citizen science provide fresh approaches to understanding and addressing health risks associated with climate change. They are crucial to aid policymakers in understanding the most equitable ways to minimize the health risks of climate change. 

To minimize knowledge gaps, federal policy needs to ensure that there is a comprehensive strategy to drive a climate change and health research agenda and adequate resources to sustain it. Without federal policy that increases funding directed toward this effort, coordinates resources and activities, and continues to build capacity, research will remain stymied. We encourage the public health policy and research community to take advantage of this opportunity to advance the state of research by prioritizing the application of the methods and tools discussed here to climate change and health. Doing so may provide critical knowledge to further motivate and improve public health-related policies on climate change.
